# Supplementary material for: Seed-Fill-Shift-Repair: A redistricting heuristic for civic deliberation
Source: PLoS One. 2020 Sep 11;15(9):e0237935. doi: 10.1371/journal.pone.0237935 (PMC7485828; doi:10.1371/journal.pone.0237935)
Supplement: Attachment — Submitted filename: response-to-reviewers.pdf [file pone.0237935.s002.pdf]

Response to Reviewers

The Authors

July 18, 2020

Responses in yellow. In addition we are providing, as requested, diff.pdf, which shows the differences between the file originally submitted and the one newly submitted here.

This document answers to the editor's request to include the following item when submitting our revised manuscript:

> A rebuttal letter that responds to each point raised by the academic editor and reviewer(s). This letter should be uploaded as separate file and labeled 'Response to Reviewers'.

1 Editor's Comments and Requirements

> Journal Requirements:
>
> When submitting your revision, we need you to address these additional requirements.
>
> 1. Please ensure that your manuscript meets PLOS ONE's style requirements, including those for file naming. The PLOS ONE style templates can be found at
>
> `https://journals.plos.org/plosone/s/file?id=wjVg/PLOSOne_formatting_sample_main_body.pdf` and
>
> `https://journals.plos.org/plosone/s/file?id=ba62/PLOSOne_formatting_sample_title_authors_affiliations.pdf`

/1/ We believe we are in compliance.

> 2. We note that you have stated that you will provide repository information for your data at acceptance. Should your manuscript be accepted for publication, we will hold it until you provide the relevant accession numbers or DOIs necessary to access your data. If you wish to make changes to your Data Availability statement, please describe these changes in your cover letter and we will update your Data Availability statement to reflect the information you provide.

/2/ We do not wish to alter our Data Availability statement and have provided DOIs for accessing our code and data. See the "Supporting information" section.

3. Please upload a copy of Supporting Information Files which you refer to in your text on page 19.

/3/ There are now five items listed in the "Supporting information" section. S1, the source code zipped up, is now being submitted directly to PLOS One with this document. S2–S5 have DOIs and are hosted on Zenodo.

2 Reviewer's Responses to Questions

Comments to the Author

1. Is the manuscript technically sound, and do the data support the conclusions?

The manuscript must describe a technically sound piece of scientific research with data that supports the conclusions. Experiments must have been conducted rigorously, with appropriate controls, replication, and sample sizes. The conclusions must be drawn appropriately based on the data presented.

Reviewer #1: Partly

Reviewer #2: No

2. Has the statistical analysis been performed appropriately and rigorously?

Reviewer #1: No

Reviewer #2: No

/4/ The goal of the paper is to present an algorithm and demonstrate its functioning. This is NOT PRIMARILY a study that uses data to test hypotheses. Instead, we are in effect contributing a tool for use in science, for finding redistricting plans. We demonstrate that the tool works and document it in detail. In the spirit of these two questions, however, we have considerably lengthened the "Computational results" section, adding three new subsections, one in which we report on generating plans for five different states, using the 2018 ACS data, another in which we report statistics from sampling the corpus of 11,206 plans for Pennsylvania and present a statistical analysis of the reliability of SFSR in finding feasible plans. Further, we have added pertinent descriptive statistics throughout the paper to augment the discussion of the solutions. Finally, at the end of the "Computational results" section we have added a statistical analysis that highlights the high reliability of SFSR on the cases described in the paper.

3. Have the authors made all data underlying the findings in their manuscript fully available?

The PLOS Data policy requires authors to make all data underlying the findings described in their manuscript fully available without restriction, with rare exception (please

refer to the Data Availability Statement in the manuscript PDF file). The data should be provided as part of the manuscript or its supporting information, or deposited to a public repository. For example, in addition to summary statistics, the data points behind means, medians and variance measures should be available. If there are restrictions on publicly sharing data?e.g. participant privacy or use of data from a third party?those must be specified.

Reviewer #1: No

Reviewer #2: Yes

/5/ In our original submission we indicated that we would make our code and the data it generated—the redistricting plans discussed in the paper—fully available without restriction. In our cover letter to the editor we also indicated that our files were too large for PLOS and so we would need to find an alternative host for the files. We have done this and indicated the pertinent DOIs in the "Supporting information" section of the paper.

4. Is the manuscript presented in an intelligible fashion and written in standard English?

PLOS ONE does not copyedit accepted manuscripts, so the language in submitted articles must be clear, correct, and unambiguous. Any typographical or grammatical errors should be corrected at revision, so please note any specific errors here.

Reviewer #1: Yes

Reviewer #2: Yes

/6/ We have in addition repaired a number of typos in expressive infelicities we found during revision.

3 Review Comments to the Author

3.1 Reviewer #1

The paper suggests a heuristic called SFSR based on constraint satisfaction for creating electoral redistricting plans for the state of Pennsylvania. The problem addressed is relevant and the suggested method adopts an interesting strategy for its solution. I have just a few items I would like to address before I am fully convinced that this article has been published on PLOS-ONE.

1. INTRODUCTION

For motivation, it is important to give a more detailed context of the problem. To this, it would be useful to include a paragraph that briefly explains the functioning of the American electoral system, how the seats are divided in the representatives' house, as well the presentation of concepts associated with the theme (e.g. electoral precinct, gerrymandering). As an example, the term gerrymandering (line 142) is used without its

concept and implications being previously described. For a better understanding, a figure could illustrate examples of favoring situations caused by gerrymandering.

/7/ Thank-you, this is nice suggestion. We have added to the Introduction a substantial description of the peculiar American situation with regard to redistricting in the context of most districted offices in the United States being single-member districts decided by a majoritarian electoral system, a definition of gerrymandering from a recent U.S. Supreme Court case, and a series of tables (1–4) with discussion that vividly show why it matters who draws the redistricting lines.

3. METHODS

As informed by the authors, the source code and results were not made available due to the size of the files. As a suggestion, the "ResearchGate" portal allows the creation of DOI and storage of files up to 512 MB.

/8/ Thank-you for the suggestion. After investigation we also looked at Figshare `https://figshare.com` and Zenodo `https://zenodo.org`. We have decided to use Zenodo. The DOIs we indicate in the "Supporting information" section of the paper are hosted there.

The method was presented from the code perspective but as it is not available, its understanding was more laborious. For reproduction purposes, I think the most appropriate format would be the description of the algorithm in pseudocode, which contains the actions of the method in a generic way and independent of language or implementation strategy. Including a flowchart that represents the steps of the method would also assist in its comprehension. The details of the implementation are important and may be contained in a file that accompanies the code itself. It would be interesting if the files with input data (lines 429 to 432) were also made available.

For higher assertiveness, I see that it would be more appropriate for the detailing of the components described in the section "The SFSR algorithm in detail" to be done in the Methods section itself in the respective procedure. The operation of the "Contiguity Checking" procedure was not very clear and deserves further details, especially about the matrix reduction strategy described in lines 542 to 545.

/9/ We have elected to add pseudocode (Algorithm 1) and not a flowchart. See Algorithm 1 in the current version of the paper. We have also refactored the code somewhat (without changing the underlying SFSR algorithm). We document the new organization in the paper per the reviewer's suggestions.

COMPUTATIONAL RESULTS / DISCUSSION

I miss details about the development and experiment environment, with information about versions of the adopted language, libraries, operating system, and hardware specifications (basically processor and memory).

/10/ We added additional implementation details for the program and the main language and packages used. As mentioned in the “Implementation” section, the run times reported here were measured on a workstation, as specified. In our substantial experience with regular laptop/desktop configurations, the times are not significantly longer. We believe we have been fully responsive to this request, although the information appears in several places.

Although the authors gave an idea of the time consumed by the method (line 587), I think that this measure could be better explored. Considering that the method adopts repeated solution adjustment strategies, it would be interesting to describe information about the time spent in each step of the method, to have an idea of the implications related to the convergence time to the viable solution.

/11/ In addition to the previously mentioned implementation details, we added runtime information for solutions calculated for several states and presented the results as boxplots. This gives a good overview on expected runtime distributions. Furthermore, we also added the information that on average, both the shift and the repair procedures make up about 50% of the overall runtime each.

In the figures presented, it was not clear certain notes described in the text. For example, in Figure 1 (lines 613-614) it is not evident where splits occurred in the counties; as well as in Figure 2 (lines 632 and 633) the districts with the highest incidence of minorities. Highlighting the changes in the figure can make the benefits of the proposed method clearer.

/12 part 1/ We have dropped the Figure 1 of the previous draft, referred to in the reviewer’s comment above because it is not possible to display the requested information on a graphic of a size suitable to a journal article. The information can be seen with a GIS and lots of zooming and panning, but even there not all that easily on a single screen. In consequence, we dropped the figure and simply report the results in the text. For interested readers, the information is extractable from the data files we have posted. There are 67 counties in Pennsylvania. See just below what the extracted data on split counties looks like using our analytics scripts:
While it is easy enough to produce a large table in LaTeX for the paper, we really doubt the usefulness of doing this.
What we have done is to provide a Jupyter Lab notebook that can calculate the county split information for any given plan. The file is CountySplitsCalculator.ipynb and we have indicated it as S4 Files with DOI 10.5281/zenodo.3911273.
Example output for the plan ’df2020-01-31x14x56x15x610108pc0.01x1618.csv’ which we discuss in Figure 5, is given below this comment. It’s a Python dict.

```
{'Allegheny County': {9, 11, 14},
 'Cambria County': {2, 16},
 'Delaware County': {3, 5},
```

```
'Clinton County': {16, 17},
'Crawford County': {10, 16},
'Adams County': {2, 4},
'Huntingdon County': {2, 16},
'Clearfield County': {16},
'Lawrence County': {10},
'Columbia County': {17},
'Monroe County': {1, 13},
'York County': {4, 12},
'Forest County': {16},
'Wyoming County': {13},
'Montour County': {17},
'Jefferson County': {16},
'McKean County': {16},
'Erie County': {10},
'Tioga County': {16},
'Greene County': {9},
'Perry County': {2, 4},
'Fayette County': {9},
'Bedford County': {2},
'Berks County': {12, 15, 17},
'Bucks County': {1, 6, 8, 15},
'Westmoreland County': {2, 9, 11},
'Chester County': {3, 5, 12, 15},
'Dauphin County': {4, 12, 17},
'Potter County': {16},
'Fulton County': {2},
'Sullivan County': {13},
'Elk County': {16},
'Venango County': {10, 16},
'Schuylkill County': {1, 4, 15, 17},
'Mifflin County': {2, 16, 17},
'Lehigh County': {1, 15, 17},
'Lycoming County': {16},
'Warren County': {16},
'Lackawanna County': {13},
'Bradford County': {13, 16},
'Carbon County': {1, 17},
'Washington County': {9},
'Lebanon County': {17},
'Beaver County': {10, 14},
```

```
'Blair County': {2},
'Union County': {17},
'Luzerne County': {1, 13, 17},
'Montgomery County': {3, 5, 6, 7, 8, 15},
'Cumberland County': {2, 4},
'Philadelphia County': {3, 7, 8},
'Northumberland County': {17},
'Somerset County': {2},
'Mercer County': {10},
'Cameron County': {16},
'Franklin County': {2},
'Armstrong County': {10, 11, 16},
'Wayne County': {13},
'Clarion County': {10, 16},
'Northampton County': {1},
'Juniata County': {2, 17},
'Centre County': {16},
'Indiana County': {2, 11, 16},
'Lancaster County': {5, 12, 15, 17},
'Susquehanna County': {13},
'Pike County': {13},
'Butler County': {10, 11, 14},
'Snyder County': {17}}
```

/12 part 2/ The Fig 2 of the original submission is Fig 4 in the revised submission. We have added text to the figure's caption to indicate the minority districts in question and some information about them.

Table 2 needs to be described in more detail, it was not clear to me what the percentages represent 25%, 50% and 75% (below min). As an example, in my understanding, 25% of the plans have 12 districts with the number of whites greater than 75% (W $\geq$ 75). Would it be this? I imagine that a scatter plot can give an overview of the characteristics of the plans obtained (the choice of metrics is at the preference of the authors).

/13/ Because of the added four tables in the Introduction, this is now table 6. We have modified the explanatory text that sits below the table, greatly expanding it in order to provide an explicit and we hope utterly clear interpretation of the meaning of the table.
We thank the reviewer for pointing out the need to give this clarification and very much agree that it is needed and useful here.

The experiments were based on a single dataset. I think it is important to consider

other datasets for greater variability of scenarios, for example, different states or years. An interesting analysis would be to compare a redistricting obtained by the SFSR with another that has adopted in a real circumstance.

/14/ We added, using our SFSR code, 1000 plans each for 5 states using a different year of the ACS data, and discussed this in a new subsection of the “Computational results” section, including with various descriptive statistics. Adding new states like this is unproblematic for our code, but requires more descriptive statistical work on our part. We grant the reviewer’s point, but doubt much further is to be gained in this direction, from what we have added in this revision. If the reviewer thinks otherwise, perhaps the reviewer could suggest an expansion of cases that would be sufficient.

At various points in the text, the authors make it clear that a more extensive analysis of the method is outside the scope of the work. However, I see that this item is essential to meet the scientific rigor required by PLOS-ONE. In the Discussion section, the authors raise an interesting question about random plans and the advantages of SFSR over other methods. As a way of validating what was discussed, I think it is important that the methods in question, or at least one of them, are compared with SFSR concerning the execution time and quality of the generated plan. To compare the quality of the plans, the authors could adopt one or more external measures of your choice, for example, the number of counties splits, compactness, and so on.

I think that is valid a discussion about the flexibility (or not) of the SFSR to include other types of requirements.

/15a/ We believe the reviewer has misunderstood some of the claims of the paper, for which we take full responsibility and for which we have sought to add clearer expression. If the referees have suggestions for further clarification, we would be grateful to entertain them. We would make the following points in response to the referee's remarks.

(1) In our discussion of random plans, we are certainly *NOT* claiming that SFSR creates random plans. Like other algorithms, it uses randomization, but that is a very different thing than being able to draw a random sample from the population of feasible plans. Our remarks in the paper are critical of claims to the contrary. These claims have been made by the authors we cite, who have presented them in court without success (they were rejected by the court and we think correctly so). Our stance here is an indirect one, aimed at keeping things polite and not giving offense unnecessarily. Arguably, SFSR has the strongest claim of the published methods to being able to produce a random corpus of plans, and we are directly asserting that it cannot do this, just as the other algorithms we discuss cannot do this. So there is no claim being made here that SFSR is better than what's already out there.

(2) Yes, it would be a fine thing to compare SFSR with other methods of generating large corpora of redistricting plans. That task, however, huge and is beyond the scope of the present paper. That being said, this is one area where research is headed – see the cited study by Fifield and colleagues (2019) in our revised version of the article for the first assessments in this area – and one that we hope to participate in future research projects. The Fifield cite is [63] in the paper:

62. Fifield B, Higgins M, Imai K, Tarr A. Automated Redistricting Simulation UsingMarkov Chain Monte Carlo. Journal of Computational and Graphical Statistics.2020;doi:https://doi.org/10.1080/10618600.2020.1739532

Also, we address this issue directly in several places now, most explicitly in the following passage.

> It is also beyond the scope to run computational comparisons of SFSR with the algorithms discussed in "Related work," an aim which in any event is further impeded by the lack of publicly available code implementing many of these algorithms, even for results submitted as part of redistricting litigation. This state of affairs is commented on by [84], and we join them in calling for "open and reproducible development of tools for redistricting."

A main purpose of our submitting this paper to PLOS One (an open access journal) and opening up our code for public use has been to promote these sorts of comparative studies.

/15b/ (3) Comparison of alternative ways of generating large corpora of plans is a subtle matter. As emphasized in the paper, there is no generally agreed upon view of what constitutes a good plan in the public's interest. Hence, our move to create corpora of feasible plans. This is, as we discussed in the literature review, an unusual if not unique stance. It certainly would be possible and interesting to compare SFSR's plans with plans produced by an optimizing heuristic that worked on, say, compactness or county splits. But: (i) As we have said, we do not have access to such code in peer reviewed venues, if it even exists (our literature review is comprehensive). (ii) Comparison of an optimization approach with a constraint satisfaction approach would have genuine, but limited value, because any optimization approach will presumably be pretty good at finding solutions that fare well on its objective function and not so good on finding solutions that are not valued in its objective. But as we document at length in the paper, what the proper objective is is unclear and no one has succeeded in presenting an effective multi-objective algorithm for finding large numbers of high quality plans. Nothing close to this exists. In consequence it will be necessary to proceed in a piecemeal fashion, but that can't happen until researchers develop algorithms and make their code public so that comparisons can be made.
(4) We are not claiming that SFSR produces great plans. What a great plan is isn't well defined so we go for constraint satisfaction and encourage subsequent exploration and discovery. What we do claim, and demonstrate, is that SFSR can reliably generate lots of plans, plans that are feasible. SFSR is a start. How to support further discovery of new and more attractive plans is a matter for future research.
(5) Granting without hesitation that, were the means to hand, it would be good to compare the plans generated by different algorithms, we do want to make a more subtle point. All approaches are going to be heuristics. Seeking the best heuristic is an iffy endeavor, if only for reasons of the No Free Lunch Theorem. A more nuanced approach would recognize that different heuristics will search different regions of the solution space (because they are not in fact sampling randomly). For this reason, conceptually thinking of designing a hyper-heuristic makes a lot of sense. Given a number of plan generating heuristics at its disposal, what would a smart hyper-heuristic learn about allocating computational effort across the several methods? Great question, grand challenge, doable some day, but not now. What this paper does is provide open-source code for us and other researchers to begin to explore these questions.
(6) Finally, on comparisons with other methods, we have added descriptive statistics throughout about the plans discovered and about the computations to create them. These statistics are, to the best of our knowledge utterly unavailable for other corpora of redistricting plans. Again, our hope is that in publishing this paper we encourage work that affords comparison of different corpora of plans and different ways of creating them.

3.2 Reviewer #2

The present manuscript discussed on the used of Seed-Fill-Shift-Repair (SFSR) districting algorithm. However, there are few points that the authors may consider in order to improve this manuscript:

1. The abstract is too technical and not adhere overview of the problem. The authors had straightforwardly describe the method, which at some point could be good in some readers, but it would be better if the authors can explain the general problem that they need to solve;

/16/ We have revised and lengthened the abstract in order to tell a more complete and accessible, but still concise, story on what the paper is about. We hope we have met the sense of the reviewer's request.

2. The explanation about the method is very intuitive, which is very good for those who are familiar in this field like myself, but it would be better if the authors can present some other ways to present the proposed method (i.e. pseudocode, flowchart). This is very helpful especially when explaining the experimental setup

/17/ See response /9/.

3. The results are very limited and need more proof i.e. using statistical analysis

/18/ The "Computational Results" section has been greatly expanded, with both descriptive and confirmatory statistics, where appropriate. Three entirely new subsections have been added: "Implementation", "Congressional Districting for U.S. States" in which we extend the results and analyses to five states and using an updated ACS data set, and "Effect of Solution Set Size" in which we explore the effect of solution set size on finding solutions with less typical characteristics and we assess the reliability (which is very good for the cases examined) with which SFSR finds feasible solutions. See also /15 a–b/ above, especially (6).
